# Multipoint *Kras* oncogene mutations potentially indicate mucinous carcinoma on the entire spectrum of mucinous ovarian neoplasms

**DOI:** 10.18632/oncotarget.13449

**Published:** 2016-11-18

**Authors:** Yi-Ju Lee, Ming-Yung Lee, Alexandra Ruan, Chi-Kuan Chen, Hao-Ping Liu, Chau-Jong Wang, Wan-Ru Chao, Chih-Ping Han

**Affiliations:** ^1^ Institute of Biochemistry, Microbiology and Immunology, Chung-Shan Medical University, Taichung, Taiwan; ^2^ Department of Pathology, Chung-Shan Medical University Hospital, Taichung, Taiwan; ^3^ Department of Statistics and Informatics Science, Providence University, Taichung, Taiwan; ^4^ Stanford School of Medicine, Stanford, California, USA; ^5^ Department of Pathology; Laboratory Medicine, Mackay Memorial Hospital, Taipei, Taiwan and Department of Medicine, Mackay Medical College, Taiwan; ^6^ Department of Veterinary Medicine, College of Veterinary Medicine, National Chung Hsing University, Taichung, Taiwan; ^7^ Institute of Biochemistry, Microbiology and Immunology, Chung Shan Medical University, Taichung, Taiwan; ^8^ Department of Medical Research, Chung Shan Medical University Hospital, Taichung, Taiwan; ^9^ Department of Pathology, Chung-Shan Medical University and Chung Shan Medical University Hospital, Taichung, Taiwan; ^10^ Department of Obstetrics and Gynecology, Chung-Shan Medical University and Chung-Shan Medical University Hospital, Taichung, Taiwan

**Keywords:** mucinous adenoma (MA), mucinous borderline tumor (MBT), mucinous carcinoma (MC), anti-epidermal growth factor receptor (anti-EGFR), Pathology Section

## Abstract

*Kras* mutation is a common phenomenon in many human neoplasms. We aimed to assess the *Kras* mutational status along the histological continuum from normal ovaries to the development of benign, borderline and malignant ovarian mucinous neoplasms. We analyzed 41 cases of malignant, 10 cases of borderline, 7 cases of benign mucinous ovarian tumors and 7 cases of normal ovarian tissue. The prevalence of *Kras* mutations in the normal ovary was 0.00% (*n*=0/7), while the prevalence in benign, borderline and malignant mucinous neoplasms was 57.14% (*n*=4/7), 90.00% (*n*=9/10) and 75.61% (*n*=31/41), respectively. Multiple *Kras* mutations were detected in 6 cases of mucinous carcinoma, including 5 double mutations with G13D/V14I (*n*=1), G12V/G13S (*n*=1), G12D/G13S (*n*=3) and one triple mutation with A11V/G13N/V14I (*n*=1). We identified six cases with 3 novel *Kras* mutations not previously described in the COSMIC database, which included A11V (*n*=3) and V14I (*n*=2) in mucinous carcinomas, and A11T (*n*=1) in a mucinous borderline tumor. In conclusion, *Kras* mutation appears to be one of the imperative events in the ovarian mucinous *adenoma*–*borderline tumor*–*carcinoma* sequence, as increased numbers of *Kras* mutations have been shown to be the strongest predictor of unequivocal malignancy in ovarian mucinous neoplasms.

## INTRODUCTION

Mucinous ovarian neoplasms represent a spectrum of malignant behavior, and have benign, borderline, and malignant histopathologic variants. Their malignant potentials are correlated to their pathologic features, [1], and it is not uncommon that mucinous adenoma (MA), mucinous borderline tumor (MBT) and mucinous carcinoma (MC) components can coexist within an individual mucinous ovarian neoplasm. [2–4] Moreover, the morphological transitions from MA to MBT and from MBT to MC can easily be recognized, which supports the hypothesis of a stepwise progression through the MA-MBT-MC sequence. [5–7]

To date, it has been well established that the *Kras* oncogene plays a pivotal role in tumorigenesis. *Kras*-activating mutations often occur on exon 2, which brings about constitutive activation of the protein by increasing GDP/GTP exchange or by decreasing GTPase activity of the protein, thus triggering increased cell proliferation. [8, 9] The prevalence of *Kras* mutations seems to be highly related to tumor histology. In general, *Kras* is one of the most frequently occurring genetic abnormalities in mucinous ovarian carcinomas, and *Kras* mutations occur more frequently in mucinous *versus* non-mucinous types of ovarian neoplasms. [9–11] Furthermore, identical *Kras* mutations in adjacent MA and MBT areas of MC have reinforced the aforementioned “MA-MBT-MC sequence” hypothesis. [12, 13]

Both colorectal and mucinous ovarian neoplasms share similar histopathologic and cytogenetic characteristics. The success of anti-epidermal growth factor receptor (anti-EGFR) therapy for patients of the *Kras* non-mutation group of colorectal cancer has raised the expectation that other malignancies, such as *Kras* non-mutation group of ovarian MC can also be treated with comparable success rates. [14] In this study, we aimed to obtain more information on the spectrum of *Kras* mutations along the histological continuum from normal ovaries through benign, borderline to malignant ovarian mucinous neoplasms.

## RESULTS

### Normal ovary (*n* = 7)

Not any *Kras* gene mutation was detected in all cases of this type of ovarian tissue (*n* = 0/7). (Table 1)

**Table 1:** *Kras* oncogene status in normal ovary and various mucinous ovarian neoplasms

		Histological types of ovarian tissues				P-value
		Normal ovary	Mucinous adenoma	Mucinous borderline tumor	Mucinous carcinoma	
*Kras*	Wild	7 (100.00%)	3 (42.86%)	1 (10.00%)	10 (24.39%)	<0.001^†^, <0.001^‡^
	Mutation	0 (0.00%)	4 (57.14%)	9 (90.00%)	31 (75.61%)	

† Chi-square test

‡ Cochran-Armitage trend test

### Mucinous adenoma (MA) (*n* = 7)

**Figure 1:**
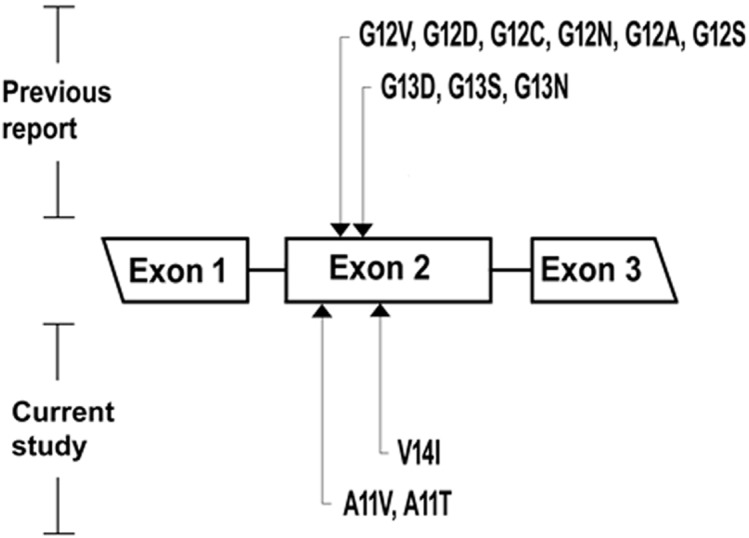
Of the mucinous ovarian neoplasms, the location of *Kras* mutation in exon 2 is presented The mutation sites reported in previous studies (codon 12, 13) are in the top, and the novel mutation sites reported in current study (codon 11, 14) are in the bottom.

The frequency of *Kras* somatic mutation was 57.14 % (*n* = 4/7) in this type of ovarian tissue. (Table 1) Codon 12 mutation was identified in one case (1/7 = 14.28%), which was identified as G12D. Codon 13 mutations were identified in three cases (3/7 = 42.86%), which presented as G13S in all three cases. No multipoint *Kras* mutations were detected. (Figure 1)

### Mucinous borderline tumor (MBT) (*n* = 10)

The frequency of *Kras* somatic mutation was 90% (*n* = 9/10) in this type of ovarian tissue. (Table 1) Codon 12 mutations were identified in six cases (6/10 = 60%), which was comprised of G12V in 1 case, G12D in 3 cases, G12C in 1 case and G12N in 1 case. Codon 13 mutations were identified in two cases (2/10 = 20%), which comprised of G13D in 1 case and G13S in 1 case. A Codon 11 mutation was identified in 1 case (1/10 = 10%), which was designated A11T. (Figure 1) No multipoint *Kras* mutations were detected.

### Mucinous carcinoma (MC) (*n* = 41)

**Figure 2:**
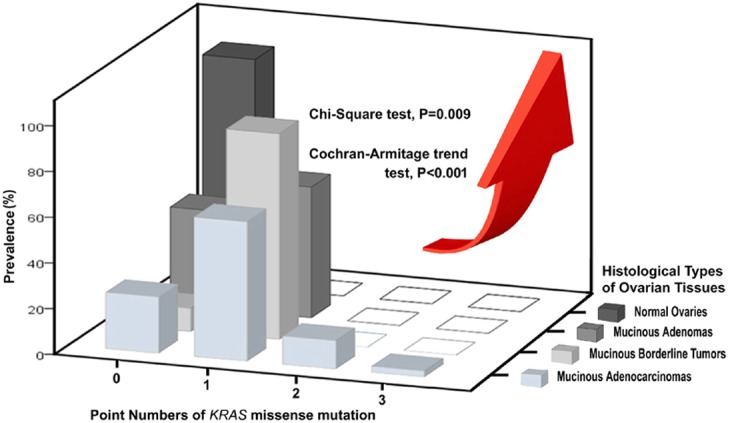
Three-dimensional histogram illustrating the associations among prevalence, point numbers of *Kras* missense mutation and 4 histological types of ovarian tissues

The frequency of *Kras* somatic mutations was 75.61% (*n* = 31/41) in this type of ovarian tissue, which consisted of both single and multipoint mutations subgroups. (Table 1) (Figure 2)

Of the single *Kras* mutation subgroup (25/41 = 60.98%), codon 12 mutations were identified in twenty cases (20/41 = 48.78%), which were composed of G12V in nine cases, G12D in eight cases, G12C in one case, G12A in one case and G12S in one case. Codon 13 mutations were identified in three cases (3/41 = 7.32%), which were all comprised of the G13S mutation. Codon 11 mutations were identified in two cases (2/41 = 4.88%), which were identified as A11V in both cases. Codon 14 mutation was not identified. (Figures 1, 2)

Of the multiple *Kras* mutations subgroup (6/41 = 14.64%), double mutations were identified in five cases (5/41 = 12.20%) and a triple mutation was identified in one case (1/41 = 2.44%). Three cases had both codon 12 (G12D) and codon 13 (G13S) mutations; one case had both codon 13 (G13D) and codon 14 (V14I) mutations; one case had both codon 12 (G12V) and codon 13 (G13D) mutations; and one case had triple codon 11 (A11V), codon 13 (G13N) and codon 14 (V14I) mutations. (Figures 1, 2) Additionally, multipoint *Kras* mutations (*n* = 2 or 3) were only recognized in MC, but were not detected in MBT, MA or normal ovarian tissue types. (Figure 2)

### Novel *Kras* mutations in mucinous ovarian neoplasms

In addition to the previously reported codon 12 and 13 aberrations which harbored 9 mutations, we identified 3 novel mutations (A11V, A11T, V14I) based on the COSMIC (Catalogue of somatic mutations in cancer) database accessed on 12/06/2016. (Figure 1)

### *Kras* mutation rates positively associated with the normal ovary-MA-MBT-MC sequence

The percentages of *Kras* mutations are significantly different among the 4 histological types of ovarian tissues. (Chi-Square test, *p* < 0.001) (Table 1) The prevalence of *Kras* gene mutations also significantly increased when assessed along the sequence order of: the histologically normal ovary (*n* = 0/7, ratio = 0.00%) through MA (*n* = 4/7, ratio = 57.14%), MBT (*n* = 9/10, ratio = 90%) to MC (*n* = 31/41, ratio = 75.61%) (Cochran-Armitage trend test, *p* < 0.001). (Table 1)

### *Kras* mutation rates are positively associated with the missense point numbers (*n* = 0-3) across 4 histologic types of ovarian tissues (normal ovaries, MA, MBT, MC)

There are significant differences among the 4 types of *Kras* mutation with different missense point numbers (*n* = 0-3). (Chi-Square test, *p* = 0.009) (Figure 2) Their prevalence stratified by *Kras* missense mutation numbers (*n* = 0-3) show a significantly increasing trend in the sequence order from the histologically normal ovaries through MA to MBT to MC (Cochran-Armitage trend test, *p* < 0.001). (Figure 2)

## DISCUSSION

Mutation analysis of the *Kras* oncogene has now been established as a predictive biomarker in colorectal cancer, which signifies that wild-type *Kras* should respond to anti- EGFR treatment. Previous research has resulted in promising results. Sato N et al has reported that cetuximab inhibited the growth of ovarian MC cell lines which lacked the *Kras* gene mutation, but did not inhibit the growth of the other ovarian MC cell lines carrying the *Kras* gene mutation. [15] This encouraging finding prompted us to investigate the *Kras* mutation status and evaluate the possible therapeutic implications of anti-EGFR treatment for patients with advanced ovarian MC.

Limited information exists to address *Kras* status across different types of mucinous ovarian neoplasms, as a standard *KRAS* mutation detection assay was not established until recently. Cuatrecasas M et al (1997) found that the frequency of *Kras* mutations in codons 12 and 13 was lower in benign (58.33%, *n* = 35/60) than in borderline (86.36%, *n* = 19/22) or malignant mucinous ovarian tumors (84.61%, *n* = 11/13) by polymerase chain reaction (PCR) and restriction fragment length polymorphisms (RFLP). [16] Auner Vet al (2009) stated that the rate of *Kras* mutations in codon 12 and 13 increased from 50% (*n* = 3/6) in MBT to 60% (*n* = 15/25) in MC by GeneStix biochip platform. [17] Anglesio MS et al (2013) reported that *Kras* mutation regions encompassing codons 12 and 13 were higher in borderline (78.79%, *n* = 26/33) than in malignant mucinous ovarian tumors (43.66%, *n* = 31/71) by the Sanger-sequencing method. [18] Anglesio MS et al (2015) applied next generation sequencing (targeted deep sequencing method) to the same cohort from the previous study and re-counted *Kras* mutation data. Their results showed that *Kras* mutation remained higher in MBT (92.3%, *n* = 24/26) than in MC (64.9 %, *n* = 24/37). [19]

The assay used in this study, direct sequencing, allows for the detection of all mutations and is now considered to be the gold standard for mutation detection. [20] Several additional techniques are available, which have been developed since the location of *Kras* mutations is discrete. [21, 22] FemtoPath *Kras* Mutation Screen Kit is a PCR-based test using novel and proprietary primers which can specifically and sensitively amplify somatic mutations in the *Kras* gene and suppress the amplification of wild-type *Kras* gene in human genomic DNA. [23]

In this study, we used the *Kras* mutant-enriched PCR Kits (FemtoPath^®^) with subsequent direct sequencing method to analyze the mutation status of *Kras* exon 2, codons 11-14 in 65 ovarian tissues, including 7 normal ovarian tissues and 58 mucinous neoplastic tissues (7 MAs, 10 MBTs and 41 MCs). Of the former group, mutations in codons 11-14 were not detected in any of the samples. Of the 58 mucinous ovarian neoplasms, codon 12 mutations were detected in 53.45% (*n* = 31), comprised of 1 MA, 6 MBTs and 24 MCs; codon 13 mutations were detected in 24.14% (*n* = 14), comprised of 3 MAs, 2 MBTs, and 9 MCs; codon 11 mutations were detected in 6.90% (*n* = 4), comprised of 0 MA, 1 MBT and 3 MC; and codon 14 mutations were detected in 3.45% (*n* = 2), comprised of 0 MA, 0 MBT and 2 MCs. Thus, we re-confirmed that codon 12 was the most common location of *Kras* mutation in both MBTs and MCs, and codon 13 was the most common location of *Kras* mutation in MAs. Our results were generally compatible with the aforementioned four studies. [16–19] Furthermore, we identified that both codons 11 and 14 were less common locations of *Kras* mutations in MBT and MC only.

Our results identified 9 amino acid substitutions (G12V, G12D, G12C, G12N, G12A, G12S, G13D, G13S, G13N) at codons 12 and 13 in exon 2. We have also found 3 unusual missense mutations at codons 11 and 14 (A11V, A11T and V14I), which have never been reported in the COSMIC *Kras* mutations database in ovarian tumors, and thus should be considered novel oncogenic mutations. We suggest the introduction of both codons 11, 14 as hotspots for routine *Kras* mutational analysis in patients with different histologic types of mucinous ovarian neoplasms, rather than focusing only on codons 12 and 13. All above-mentioned mutations were determined to be somatic since the sections of 7 normal ovarian tissues showed wild type sequence. The functional impact of the novel mutations on KRAS protein requires further bioinformatics tools and molecular models. We detected one of the novel mutations (A11T) in 1 case of 7 MBTs and the other two (A11V, V14I) in 4 cases of 41 MCs. Our discovery of novel codon 11 and 14 mutations in exon 2 that are, so far, unique to Taiwanese patients may be attributed to the genetic variations amongst different racial/ethnic groups. Alternatively, it may again be related to the lack of clinical studies on mutations other than those at codon 12 and 13 and could, in the future, prove to be frequent and non-racially restricted. Even though the sample size in this study was small, further large scale studies of various ethnicities and bio-functional analysis of the discovered novel mutations as well as their possible role in ovarian mucinous carcinogenesis merit further investigation.

Missense mutations in codons 12 or 13 are the most frequent mutations in *Kras* oncogene, but multipoint mutations in other codons can also develop. Little data exists on multipoint *Kras* mutations regarding their frequency or the codons and amino acids affected. Of all 41 mucinous carcinomas, double mutations (G12V/G13D) were identified in only 5 cases (12.20%), and a triple mutation (A11V/G13N/V14I) was detected in only 1 case (2.44%). We were very interested to find that multipoint *Kras* mutations only existed in MC, representing 14.44% (*n* = 6/41) of cases; but did not exist in MA (*n* = 0/7) or MBT (*n* = 0/10). The point number of *Kras* mutations (*n* = 0-3) is positively associated with the increase of malignant potentials along the normal ovary-MA-MBT-MC sequence. (Cochran-Armitage trend test, *p* < 0.001) (Figure 2) Our data revealed that multipoint *Kras* mutations (*n* = 2, 3) can be regarded as a possible indicator of MC among the 4-histologic types of ovarian tissues.

Due to the presence of additional mutations, we also imagined that common *Kras* mutations in the neighboring MA and MBT areas might have been carried over into the adjacent MC parts. However, it is unclear at present whether the multipoint *Kras* mutations represent biologically molecular events or whether they designate the concurrence of different neoplastic clones within a given tumor.

Clinical trials of EGFR inhibition in ovarian cancer have been disappoint­ing to date [24]. However, the emerging novel EGFR inhibitors will create alternative therapeutic opportunities. Accurately determining *Kras* status in ovarian MC is advisable to select potentially appropriate candidates for newly developed anti-EGFR drug therapies in clinical trials. [25]

## CONCLUSIONS

The frequency of activating mutations of *Kras* at codons 11, 12, 13 and 14 indicate that *Kras*/MAPK is a crucial pathway in the carcinogenesis of ovarian mucinous tumor. Our discovery of 3 novel *Kras* mutations (A11T, A11V, V14I) that are, so far, unique to Taiwanese patients may be attributed to racial/ethnic differences. Our study showed that the number of *Kras* missense mutations is positively associated with the increase of malignant potential along the normal ovary-MA-MBT-MC categorical sequence. Increasing the missense points of *Kras* oncogene mutation seems to result in stronger malignant potential. Additionally, multipoint *Kras* mutations (*n* = 2 or 3) can be regarded as a possible indicator of the ovarian mucinous carcinoma.

## MATERIALS AND METHODS

### Specimen

Sixty-five Taiwanese cases were selected from formalin-fixed, paraffin-embedded oophorectomy tissue blocks and retrieved from the archives of the Tissue Bank of the Clinical Trial Center, Chung-Shan Medical University Hospital. The tissue samples included 4-histological types of ovarian tissues, which consisted of 7 normal ovaries, 7 MA, 10 MBT and 41 MC. All of the donors' identities have been permanently deleted. The research was conducted according to International Council on Harmonization (ICH) guidelines and compliant with all applicable regulations for the protection of human subjects for research, including review and approval by the Institutional Review Board of the Chung-Shan Medical University Hospital.

### Microdissection, DNA extraction and *Kras* mutation detection

Two board-certified pathologists (CP Han and WR Chao) reviewed all hematoxylin-and-eosin-stained (H & E) slides for these cases. For the normal ovarian tissues, the sections of normal ovary containing cortical surface epithelia and epithelial lining of small inclusion cysts were sent for DNA extraction. For the neoplastic ovarian tissues, a representative H & E section was assessed and needle microdissection was performed on subsequent 10-mm sections to obtain a high percentage of tumor cells regardless of fibroblast cell components. DNA was extracted using the QIAamp^®^ DNA FFPE Kit (Qiagen, Vialencia, CA, USA) according to manufacturer instructions. The candidate gene (*Kras*) mutation status was analyzed using the *Kras* mutant-enriched kit (FemtoPath^®^) according to manufacturer recommendations. Briefly, the self-competitive primer comprises a 5′-competing domain and a 3′-elongating domain. The 3′-elongating domain serves as a forward primer for a PCR-based amplification of the sample nucleic acid, and the sample nucleic acid with the mutation sequence is preferentially amplified over the wild type sequence.

### Sequencing

PCR products were sequenced in reverse direction with primers of *Kras* mutant-enriched PCR kit (FemtoPath^®^) by Sanger sequencing (ABI3730XL, Genomics^®^). The sequence was interpreted by visual inspection of the DNA sequence generated by Chromas software (Technelysium Pty Ltd)

### Statistical analysis

Chi-squared test was used to compare the association between mutation status and different histopathological categories. Cochran-Armitage trend test was applied to assess for a trend of positive percentages across the ordinal variables. Data were analyzed using standard statistical software (SPSS, Inc., Chicago, IL). All tests were 2-sided and the significance level was 0.05.

## References

[1] Brown J, Frumovitz M (2014). Mucinous tumors of the ovary: current thoughts on diagnosis and management. Curr Oncol Rep.

[2] Prat J (2012). New insights into ovarian cancer pathology. Ann Oncol.

[3] Mok SC, Bell DA, Knapp RC, Fishbaugh PM, Welch WR, Muto MG, Berkowitz RS, Tsao SW (1993). Mutation of K-ras protooncogene in human ovarian epithelial tumors of borderline malignancy. Cancer Res.

[4] Rodriguez IM, Prat J (2002). Mucinous tumors of the ovary. A clinicopathologic analysis of 75 borderline tumors (of intestinal type) and carcinomas. Am J Surg Pathol.

[5] Hunter SM, Gorringe KL, Christie M, Rowley SM, Bowtell DD, Campbell IG, Australian Ovarian Cancer Study Group (2012). Pre-invasive ovarian mucinous tumors are characterized by CDKN2A and RAS pathway aberrations. Clin Cancer Res.

[6] Bell DA (2005). Origins and molecular pathology of ovarian cancer. Mod Pathol.

[7] Koshiyama M, Matsumura N, Konishi I (2014). Recent Concepts of Ovarian Carcinogenesis: Type I and Type II. Biomed Res Int.

[8] Mayr D, Hirschmann A, Löhrs U, Diebold J (2006). Kras and BRAF mutations in ovarian tumors: a comprehensive study of invasivecarcinomas, borderline tumors and extraovarian implants. Gynecol Oncol.

[9] Fabjani G, Kriegshaeuser G, Schuetz A, Prix L, Zeillinger R (2005). Biochip for Kras Mutation Screening in Ovarian Cancer. Clinical Chemistry.

[10] Dobrzycka B, Terlikowski SJ, Kowalczuk O, Niklińska W, Chyczewski L, Kulikowski M (2009). Mutations in the Kras gene in ovarian tumors. Folia Histochem Cytobiol.

[11] Lengyel E (2010). Ovarian cancer development and metastasis. Am J Pathol.

[12] Mandai M, Konishi I, Kuroda H, Komatsu T, Yamamoto S, Nanbu K, Matsushita K, Fukumoto M, Yamabe H, Mori T (1998). Heterogeneous distribution of K-ras-mutated epithelia in mucinous ovarian tumors with special reference to histopathology. Hum Pathol.

[13] Garrett AP, Lee KR, Colitti CR, Muto MG, Berkowitz RS, Mok SC (2001). K-ras mutation may be an early event in mucinous ovarian tumorigenesis. Int J Gynecol Pathol.

[14] Haraldsdottir S, Bekaii-Saab T (2013). Integrating anti-EGFR therapies in metastatic colorectal cancer. J Gastrointest Oncol.

[15] Sato N, Saga Y, Mizukami H, Wang D, Fujiwara H, Takei Y, Machida S, Ozawa K, Suzuki M (2012). Cetuximab inhibits the growth of mucinous ovarian carcinoma tumor cells lacking Kras gene mutations. Oncol Rep.

[16] Cuatrecasas M, Villanueva A, Matias-Guiu X, Prat J (1997). K-ras mutations in mucinous ovarian tumors: a clinicopathologic and molecular study of 95 cases. Cancer.

[17] Auner V, Kriegshäuser G, Tong D, Horvat R, Reinthaller A, Mustea A, Zeillinger R (2009). Kras mutation analysis in ovarian samples using a high sensitivity biochip assay. BMC Cancer.

[18] Anglesio MS, Kommoss S, Tolcher MC, Clarke B, Galletta L, Porter H, Damaraju S, Fereday S, Winterhoff BJ, Kalloger SE, Senz J, Yang W, Steed H (2013). Molecular characterization of mucinous ovarian tumours supports a stratified treatment approach with HER2 targeting in 19% of carcinomas. J Pathol.

[19] Mackenzie R, Kommoss S, Winterhoff BJ, Kipp BR, Garcia JJ, Voss J, Halling K, Karnezis A, Senz J, Yang W, Prigge ES, Reuschenbach M, Doeberitz MV (2015). Targeted deep sequencing of mucinous ovarian tumors reveals multiple overlapping RAS-pathway activating mutations in borderline and cancerous neoplasms. BMC Cancer.

[20] Keller G, Geist B, Slotta-Huspenina J, Langer R, Nagl F, Fend F, Höfler H, Perren A (2009). Novel multiple, monoallelic Kras mutations at codon 12 and 13. Int J Cancer.

[21] van Krieken JH, Jung A, Kirchner T, Carneiro F, Seruca R, Bosman FT, Quirke P, Fléjou JF, Plato Hansen T, de Hertogh G, Jares P, Langner C, Hoefler G (2008). Kras mutation testing for predicting response to anti-EGFR therapy for colorectal carcinoma: proposal for an European quality assurance program. Virchows Arch.

[22] Jimeno A, Messersmith WA, Hirsch FR, Franklin WA, Eckhardt SG (2009). Kras mutations and sensitivity to epidermal growth factor receptor inhibitors in colorectal cancer: practical application of patient selection. J Clin Oncol.

[23] FemtoPath, Menu Technology. Product, FemtoPath Kras Mutation Screen Kit.

[24] Coward JI, Middleton K, Murphy F (2015). New perspectives on targeted therapy in ovarian cancer. Int J Womens Health.

[25] Perren TJ (2016). Mucinous epithelial ovarian carcinoma. Ann Oncol.

